# Upregulation of miRNA-23a-3p rescues high glucose-induced cell apoptosis and proliferation inhibition in cardiomyocytes

**DOI:** 10.1007/s11626-020-00518-6

**Published:** 2020-11-16

**Authors:** Fang Wu, Feng Wang, Qian Yang, Yawen Zhang, Ke Cai, Lian Liu, Shuchun Li, YuanZheng Zheng, Jialing Zhang, Yiting Gui, Youhua Wang, Xu Wang, Yonghao Gui, Qiang Li

**Affiliations:** 1 Translational Medical Center for Development and Disease, Institute of Pediatrics, Shanghai Key Laboratory of Birth Defect, Children’s Hospital of Fudan University, Shanghai, 201102 China; 2 Cardiovascular Center, Children’s Hospital of Fudan University, Shanghai, 201102 China; 3 Department of Neonatology, Shanghai General Hospital, Shanghai Jiao Tong University School of Medicine, Shanghai, 201600 China; 4 Department of Emergency, Longhua Hospital, Shanghai University of Traditional Chinese Medicine, Shanghai, 200032 China; 5 Department of Cardiology, Longhua Hospital, Shanghai University of Traditional Chinese Medicine, Shanghai, 200032 China; 6 Cancer Metabolism Laboratory, Cancer Institute, Fudan University Shanghai Cancer Center, Shanghai, 200032 China

**Keywords:** miRNA-23a-3p, Myocardial injury, Hyperglycemia, H9c2, Proliferation, Apoptosis

## Abstract

Maternal hyperglycemia potentially inhibits the development of the fetal heart by suppressing cardiomyocyte proliferation and promoting apoptosis. Different studies have indicated that miRNAs are key regulators of cardiomyocyte proliferation, differentiation, and apoptosis and play a protective role in a variety of cardiovascular diseases. However, the biological function of miRNA-23a in hyperglycemia-related cardiomyocyte injury is not fully understood. The present study investigated the effect of miRNA-23a-3p on cell proliferation and apoptosis in a myocardial injury model induced by high glucose. H9c2 cardiomyocytes were exposed to high glucose to establish an in vitro myocardial injury model and then transfected with miRNA-23a-3p mimics. After miRNA-23a-3p transfection, lens-free microscopy was used to dynamically monitor cell numbers and confluence and calculate the cell cycle duration. CCK-8 and EdU incorporation assays were performed to detect cell proliferation. Flow cytometry was used to measured cell apoptosis. Upregulation of miRNA-23a-3p significantly alleviated high glucose-induced cell apoptosis and cell proliferation inhibition (*p* < 0.01 and *p* < 0.0001, respectively). The cell cycle of the miRNA-23a-3p mimics group was significantly shorter than that of the negative control group (*p* < 0.01). The expression of cell cycle–activating and apoptosis inhibition-associated factors *Ccna2*, *Ccne1*, and *Bcl-2* was downregulated by high glucose and upregulated by miRNA-23a-3p overexpression in high glucose-injured H9c2 cells. miRNA-23a-3p mimics transfection before high glucose treatment had a significantly greater benefit than transfection after high glucose treatment (*p* < 0.0001), and the rescue effect of miRNA-23a-3p increased as the concentration increased. This study suggests that miRNA-23a-3p exerted a dose- and time-dependent protective effect on high glucose-induced H9c2 cardiomyocyte injury.

## Introduction

Various embryo teratogenicity mechanisms are associated with maternal diabetes mellitus, such as heart defects and neural tube defects. The risk of congenital heart defects (CHDs) increases more than 4-fold in the offspring of mothers with pre-gestational diabetes (Wren et al. 2003; Brite *et al.* 2014; Øyen *et al.* 2016; Akbariasbagh *et al.* 2017). Maternal hyperglycemia might potentially inhibit cardiomyocyte proliferation and promote cell apoptosis during fetal heart development (Han *et al.* 2015; Su *et al.* 2016). Decreased cell proliferation and increased apoptosis are the two key factors leading to a reduction in the number of cardiomyocytes (Gutierrez *et al.* 2009; Su *et al.* 2017; Su *et al.* 2019). Immature cardiomyocytes actively proliferate in the fetuses of mammals but cease to proliferate shortly after birth. Cardiomyocyte hypertrophy can compensate for the reduction in the number of cardiomyocytes, which is manifested as cardiac hypertrophy and subsequent heart failure (Ding *et al.* 2013). Therefore, rescuing cardiomyocyte damage caused by hyperglycemia before birth is of great significance.

MicroRNAs (miRNAs) are a novel class of 18-25-nucleotide small noncoding RNAs that regulate gene expression post-transcriptionally, participate in epigenetic modification, and have been identified as key regulators of cardiomyocyte proliferation, differentiation, and apoptosis (Cimmino *et al.* 2005; Sluijter *et al.* 2010). A total of 204 miRNAs with the potential to stimulate neonatal cardiomyocyte proliferation were identified in a neonatal rat cardiomyocyte functional screen. miRNA-590-3p and miRNA-199a-3p represent the two most effective miRNAs because they can promote the proliferation of cardiomyocytes both in vitro and in vivo (Eulalio *et al.* 2012). Studies of gain and loss of function have also indicated that miRNA-133a can negatively regulate cardiomyocyte proliferation during heart development by regulating the expression of cell cycle–associated genes such as *Srf* and *Ccnd2* (N. Liu *et al.* 2008). Inhibition of miRNA-29c promotes murine P19 cell proliferation by stimulating the Wnt4/β-catenin pathway and suppressing cell apoptosis by promoting the expression of *Bcl-2* (Chen *et al.* 2016). However, the number of studies on miRNA in improving glucose-induced fetal cardiomyocyte injury is very limited. A few studies have reported that overexpression of miR-22 increases the cell viability of high glucose (HG)-treated H9c2 cells (Tang *et al.* 2018), that inhibition of miRNA-34a prevents HG-induced H9c2 cell apoptosis by increasing *Bcl-2* expression (Zhao *et al.* 2013), and that overexpression of miR-30c attenuates HG-induced cardiomyocyte hypertrophy (Raut *et al.* 2015).

miRNA-23 belongs to the miRNA-23/24/27 cluster, and research shows that it participates in cell cycle regulation, proliferation, and differentiation in various diseases (Bang *et al.* 2012). Liu *et al.* reported that miRNA-23 has an important impact on regulating the proliferation and apoptosis of vascular smooth muscle cells (VSMCs) (Liu *et al.* 2018). Oikawa and partners revealed that miRNA-23 regulates angiogenesis in vivo (Oikawa *et al.* 2018), while another study found that miRNA-23a promotes apoptosis. In contrast, an miRNA-23 inhibitor protects against oxidative stress-induced cardiomyocyte injury (Liu and Liu 2018). These findings indicate that miRNA-23 participates in the response to stress-induced cardiomyocyte damage.

HG inhibits fetal cardiomyocyte proliferation and promotes apoptosis in H9c2 cells according to previous studies (Diao *et al.* 2016; Liang *et al.* 2017; Sun *et al.* 2017). We questioned whether miRNA-23a-3p is related to the proliferation and apoptosis of HG-treated H9c2 cells. Therefore, in the present study, we used a cellular model to examine the roles of miRNAs in HG-induced cardiomyocyte injury. We explored whether miRNA-23a-3p inhibition is related to decreased proliferation and increased apoptosis of H9c2 cells cultured with HG and whether upregulation of miRNA-23a-3p can rescue HG-induced H9c2 cell injury. We confirmed that miRNA-23a-3p has a protective effect on HG-induced H9c2 cardiomyocyte injury. In addition, we found that miRNA-23a-3p can regulate cell cycle- and apoptosis-associated factors to repair injuries. Furthermore, our evidence shows that miRNA-23a-3p works in a dose- and time-dependent manner.

## Materials and Methods

### Cell culture and transfection

H9c2 rat embryo cardiomyocytes (Cell Bank of the Chinese Academy of Sciences, Shanghai, China) were maintained in Dulbecco’s modified Eagle’s medium (DMEM; Gibco, Grand Island, NY; Thermo Fisher Scientific, Inc., Waltham, MA) supplemented with 5 mM glucose, 10% fetal bovine serum (Gibco; Thermo Fisher Scientific, Inc.), and 100 U/ml penicillin/streptomycin (Invitrogen; Thermo Fisher Scientific, Inc.) at 37°C and 5% CO_2_. The cells have been identified by the Chinese Academy of Sciences. The glucose concentrations in the medium were 5 mM in the normal glucose group and 35 mM in the HG group. DMEM with 5 mM and 35 mM glucose were prepared with 50% glucose solution and glucose-free DMEM, with configuration ratios of 5:2773 and 35:2743, respectively. Cells were seeded in 96-, 12-, or 6-well plates and allowed to grow to 60% confluence and then transfected with 9 pmol, 60 pmol, and 120 pmol of miRNA-23-3p mimics or a negative control (Gene Pharma, Shanghai, China), respectively. transfection was performed using Lipofectamine RNAiMAX (Invitrogen, Carlsbad, CA) following the manufacturer’s instructions.

### Cell proliferation assay

H9c2 rat embryo cardiomyocytes were plated into 96-well plates at a density of 1 × 10^4^ cells/well, cultured overnight, and then treated with 35 mM glucose (HG concentration), 5 mM glucose (normal glucose concentration), or 5 mM glucose + 30 mM mannitol for the indicated time. After 6 h of HG treatment, the other two groups were transfected with NC mimics or miRNA-23a-3p mimics, and then all the three groups were cultured for 48 h. A Cell Counting Kit-8 (CCK-8) assay (Dojindo Molecular Technologies, Inc., Kumamoto, Japan) was used to test cell viability. Briefly, 10 μL of CCK-8 reagent and 90 μL of DMEM were added to each well of the plate, the plate was incubated at 37°C for 1.5 h, and then the optical density value was read at 450 nm using a microplate reader (Bio-Tek Instruments, Inc., Synergy 2, Winooski, VT).

### 5-Ethynyl-2′-deoxyuridine incorporation assay

Cardiomyocytes were seeded in 12-well plates at a density of 5 × 10^4^ cells/per well and cultured for 24 h before transfection with mimics and 48 h after transfection with mimics at 37°C for the 5-ethynyl-2′-deoxyuridine (EdU) incorporation assay (Click-iT® EdU Imaging Kits, Invitrogen). The cells were incubated for another 2 h after adding 10 μM of EdU to each well, fixed with 3.7% formaldehyde in PBS, and then incubated for 15 min and permeated with 0.5% Triton X-100 in PBS for 20 min at room temperature. After washing three times with PBS, the Click-iT reaction mixture was added to each well, and then the cells were incubated for 30 min and stained with Hoechst 33342 for 30 min at room temperature in the dark. The ratio of EdU-positive cells to the total number of Hoechst 33342-positive cells was calculated as the cell proliferation rate.

### Time-lapse acquisition using lens-free video microscopy

Cardiomyocytes were seeded in a 6-well plate at a density of 1.5 × 10^5^ cells/well and incubated overnight. Immediately after adding HG, they were placed under a lens-free video microscope (CYTONOTE, Iprasense, France) in a cell incubator at 37°C and 5% CO_2_.

The microscope was controlled by acquisition software, which was used to perform both time-lapse acquisition and holographic reconstruction. The cells were set to be imaged at 20-min intervals for 78 h after treatment, and the dynamics of cellular indicators between cell divisions can be measured from these figures. In addition, the duration of the cell cycle can also be measured from these figures by manually detecting cell division.

### Flow cytometry analysis

The FITC Annexin V Apoptosis Detection Kit I was used according to the manufacturer’s instructions (BD Pharmingen, Franklin Lakes, NJ). After being collected with trypsin without EDTA, the transfected cardiomyocytes were washed twice with cold PBS and then resuspended in 1X binding buffer, followed by mixing with 5 μL of FITC Annexin V and 5 μL propidium iodide for 5 min in turn at room temperature in the dark. Then, flow cytometry (BD FACSAria Cell Sorter, Chestnut Hill, MA) was used to detect cell apoptosis, each sample was prepared in triplicate, and the whole experiment was repeated three times.

### Real-time qPCR

An miRNeasy Mini(r) Kit (Qiagen, Hilden, Germany) was used to extract mRNA and miRNA. cDNAs of normal mRNAs and miRNA were generated by the FastKing RT Kit (KR118, TianGen, Beijing, China) and miRcute Plus miRNA First-Strand cDNA Kit (KR211, TianGen), respectively. Real-time qPCR was performed with SuperReal PreMix Plus (FP 205, TianGen) and the miRcute Plus miRNA qPCR Detection Kit (FP 411, TianGen) on an Exicycler 96 PCR thermal block (Light Cycler 480, Roche, Basel, Switzerland). The primer sequences are listed in Table 1. Rno-miRNA-23a-3p and rno-U6 primers were purchased from TianGen (CD201-T, Beijing, China), β-Actin and U6 were used as internal controls, and the relative expression levels of these mRNAs were calculated using the comparative cycle threshold method.

**Table 1.** Primers used in real-time qPCR analysis

Gene	Orientation	Sequence
*Ccnd1*	Forward	GAGGAGCAGAAGTGCGAAGA
	Reverse	GGCGGATAGAGTTGTCAGTGTAG
*Ccnd2*	Forward	CGATGATCGCAACTGGAAGC
	Reverse	TGGTCCGGATCTTCCACAGA
*Ccna2*	Forward	AGCAGGAAGACCAGGAGAAT
	Reverse	GGTGAAGGCAGGCTGTTTA
*Ccne1*	Forward	ACGGAGCTAGCCAGCGTAAG
	Reverse	AGAGTCGCTCCAACCTCCAA
*Ccnb1*	Forward	TCCCACACGGAGGAATCTCT
	Reverse	TCTGCAGACGAGGTAGTCCA
*Bcl-2*	Forward	AGGATTGTGGCCTTCTTTGA
	Reverse	CCTACCCAGCCTCCGTTAT
*β-actin*	Forward	CACCCGCGAGTACAACCTTC
	Reverse	CCCATACCCACCATCACACC

### Statistical analysis

Statistical analysis was performed using GraphPad Prism 8.0 (GraphPad Software Lin, La Jolla, CA), and measurement data and count data were expressed as the mean ± standard error of mean (SEM) and the median, respectively. For normally distributed data, a parametric test such as Student’s *t* test was used to evaluate differences between two groups, one-way analysis of variance (ANOVA) was used to analyze differences among multiple groups, and two-way ANOVA was used to evaluate the effects of HG and time variables on the proliferation of H9c2 cells. The non-parametric test Kruskal-Wallis test was used to analyze the cell number and confluence in different groups. The criteria for statistical significance were defined as *p < 0.05, **p < 0.01, ***p < 0.001, and ****p < 0.0001.

Since the cell numbers in each cell culture differed at time-point 0, the cell numbers at time-point *t* were normalized to those of the control sample (NG group) at time-point 0 as follows:

$$ {\hat{N}}_{t,g}\stackrel{\scriptscriptstyle\mathrm{def}}{=}{N}_{t,g}-{N}_{0,g}+{N}_{0, NG} $$

where *N*_*t,g*_ is the total number of imaged cells at time-point *t*, *g* represents different groups, and cell confluence was calculated and normalized similarly (Janicke *et al.* 2017).

## Results

### Upregulation of miRNA-23a-3p rescued the HG-induced inhibition of cell proliferation

**Figure 1.**
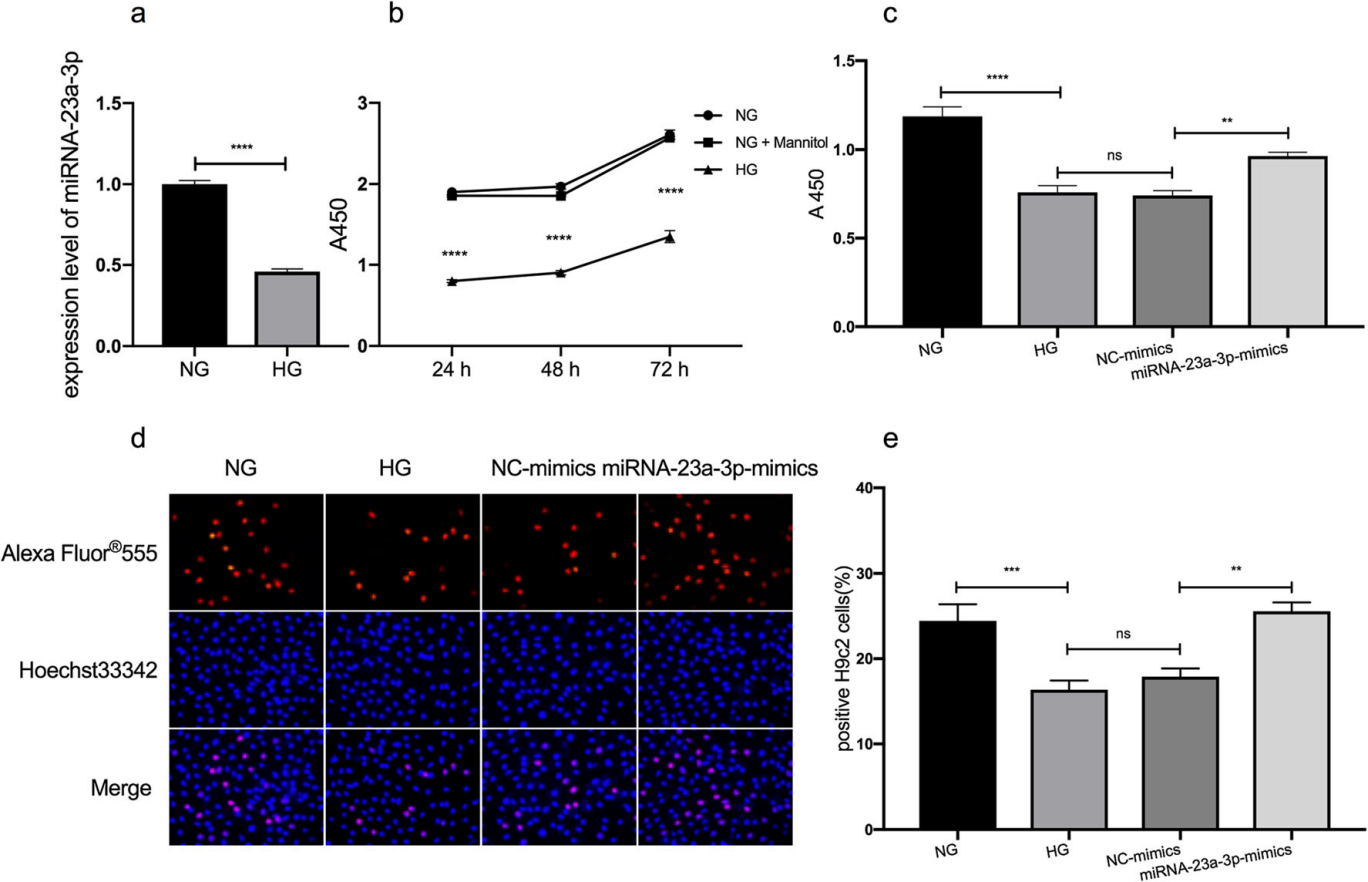
Effects of miRNA-23a-3p overexpression on cell proliferation in HG-treated H9c2 cells. (*a*) RT-qPCR to detect the expression level of miRNA-23a-3p in HG-treated H9c2 cells (****p < 0.0001 vs. NG group). (*b*) H9c2 cells were exposed to glucose at concentrations of 5 mM (NG), 35 mM (HG), and 5 mM glucose + 30 mM mannitol (NG + Mannitol) for 24, 48, and 72 h (****p < 0.0001 vs. NG group). Cell viability was assessed by a CCK-8 assay. (*c*) H9c2 cell viability was significantly increased following treatment with the miRNA-23a-3p mimics (**p < 0.01 vs. NC-mimics). (*d*) Representative images illustrating the EdU and Hoechst staining of H9c2 cells exposed to 5 mM and 35 mM glucose and transfected with NC mimics or miRNA-23a-3p mimics. (*e*) Analysis of EdU-positive cardiomyocytes by an EdU incorporation assay. The percentages of proliferative H9c2 cells were calculated (*n* > 500). Compared with the NC-mimics group, the miRNA-23a-3p-mimics group had increased EdU incorporation (**p < 0.01). CCK-8 Cell Counting Kit-8, EdU 5-ethynyl-2′-deoxyuridine, NG normal glucose, HG high glucose, NC negative control.

The expression of miRNA-23a-3p in HG-treated H9c2 cells and normal glucose-treated cells was compared by qRT-PCR in this study, and the results showed that miRNA-23a-3p in HG-treated H9c2 cells was downregulated (*p* < 0.0001; Fig. 1*a*). Since miRNA-23a-3p was downregulated in HG-treated H9c2 cells, we speculated that overexpression of miRNA-23a-3p may prevent HG-induced effects on H9c2 cells. To evaluate the effect of miRNA-23a-3p overexpression on HG-induced cardiomyocyte proliferation inhibition, H9c2 cells were treated with HG and transfected with miRNA-23a-3p mimics, and then cell proliferation was tested using CCK-8 and EdU assays.

H9c2 cardiomyocytes were first respectively treated with 35 mM glucose, mannitol, and 5 mM glucose, and cell viability was detected at different time points. As expected, cardiomyocytes exposed to 35 mM glucose showed a significant decrease in cell viability compared with the other two groups (*p* < 0.0001; Fig. 1*b*); the mannitol group was a hypertonic control group, which was used to exclude the effect of hypertonicity on cells. Then, the cells were divided into four groups, including the NG, HG, NC-mimics, and miRNA-23a-3p-mimics groups. The NC-mimics group was transfected with negative control mimics, the miRNA-23a-3p mimics group was transfected with miRNA-23a-3p mimics, and the results showed that treatment with the miRNA-23a-3p mimics significantly rescued the HG-induced cell proliferation inhibition compared with the other group (*p* < 0.01; Fig. 1*c*, CCK-8; Fig. 1*d*–*e*, EdU).

**Figure 2.**
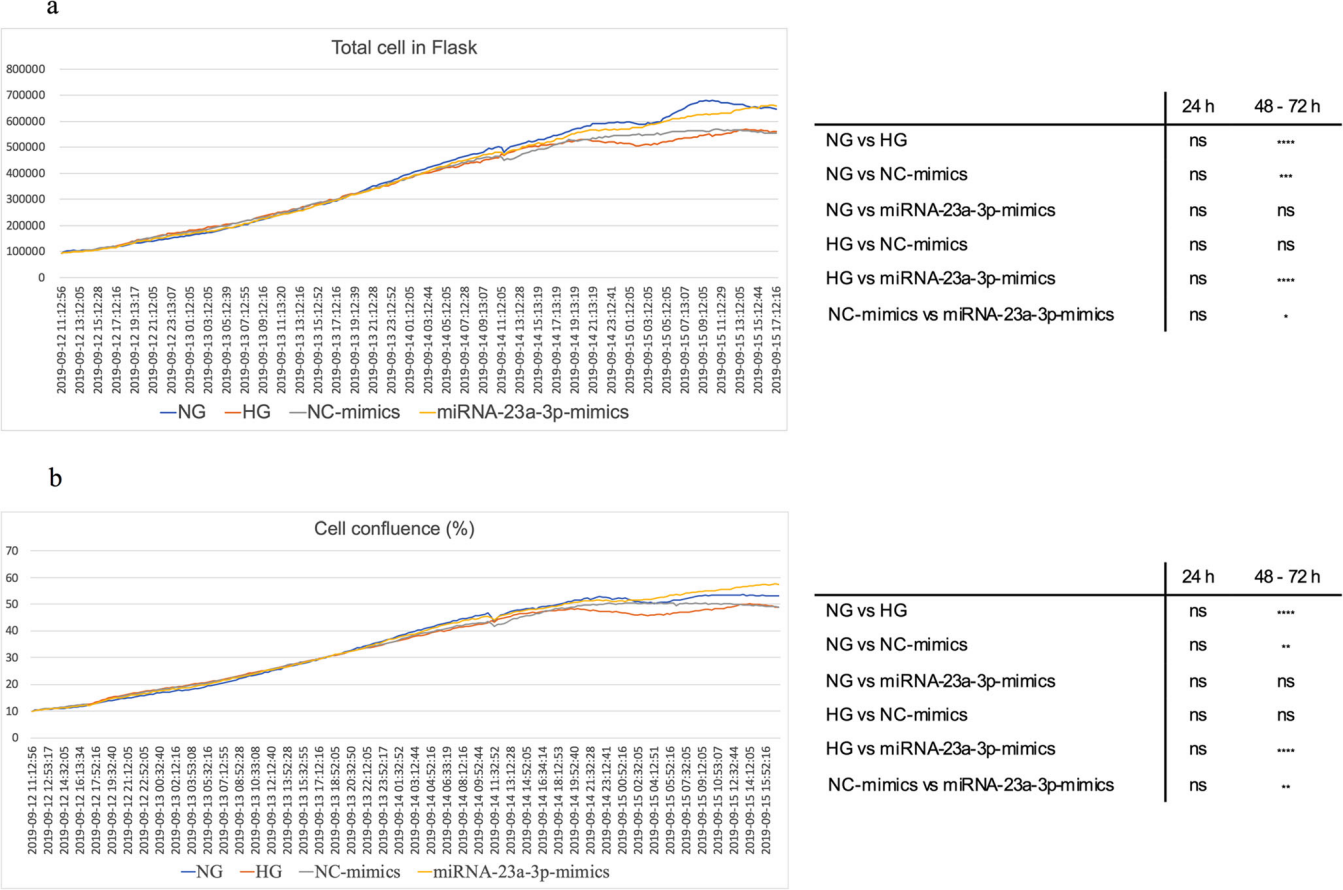
Real-time cell proliferation curves for cell cultures all curves were normalized to the NG group as described in the “Statistical analysis” section. (*a*) Cell count proliferation. (*b*) Cell confluence proliferation. The total number of cells or cell confluence in four group samples were counted every 20 min for 78 h. The statistical results are shown in the right matrix (*n* = 234). ns not significant, *p < 0.05, ***p < 0.001, ****p < 0.0001.

**Figure 3.**
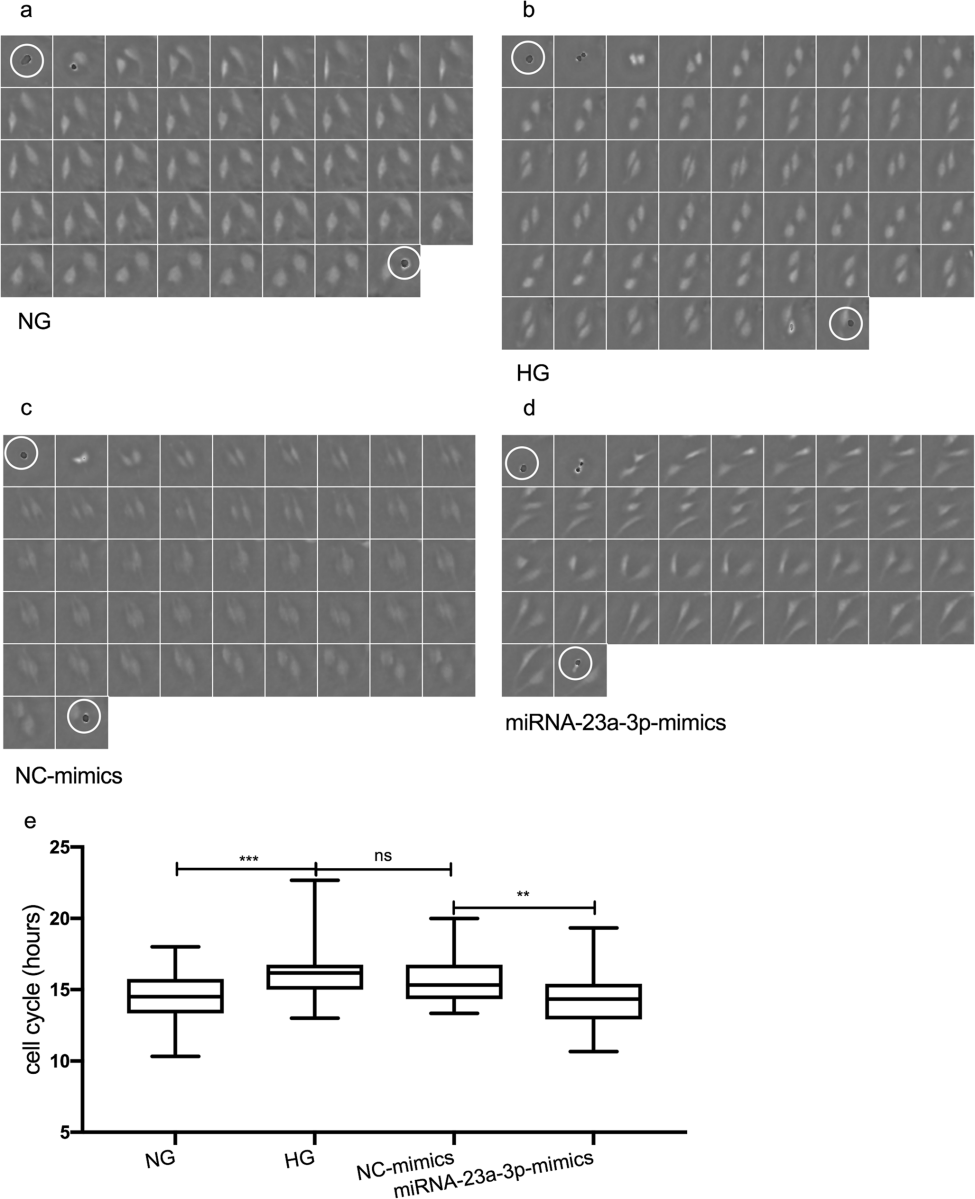
Data analysis of the cell cycle duration. Manual tracking of 200 cell cycle trajectories was performed (20-min frame interval). For example, *a*, *b*, *c*, and *d* show a single-cell trajectory evaluated with time-lapse acquisition exhibiting one cell division. (*a*) The measured cell cycle length is 14.33 h in a cell in the NG group. (*b*) The measured cell cycle length is 17 h in a cell in the HG group. (*c*) The measured cell cycle length is 15.33 h in a cell in the NC-mimics group. (*d*) The measured cell cycle length is 12.33 h in a cell in the miRNA-23a-3p-mimics group. Each cropped image is 80 × 80 μm^2^. The beginning of cell division is denoted by white circles. (*e*) Fifty cells per group were randomly tracked to calculate the cell cycle duration by detecting the occurrence of cell division in the cell track (*n* = 50). The statistical analysis of the cell cycle duration in the different groups was performed using one-way ANOVA. ns not significant, **p < 0.01, ***p < 0.001.

Lens-free microscopy was used to dynamically monitor the number and confluence of cells and calculate the average cell cycle durations of the four different groups to monitor cell proliferation dynamically. The results obtained from lens-free automatic cell counting to time-lapse acquisitions of H9c2 cell cultures over 3 d are shown in Fig. 2*a*. The proliferation assay of the miRNA-23a-3p-mimics group showed that the number of cells detected in the flask increased from 91,348 to 655,334 and that the cell confluence increased correspondingly from 9.37 to 56.95% (Fig. 2*b*). No statistically significant differences in the cell number and confluence of the four groups were observed at 24 h after transfection. However, with the emergence of cumulative effects, the cell number and confluence in the miRNA-23a-3p group were significantly increased more than those of the control group at 48 and 72 h after transfection (*p < 0.05, **p < 0.01, respectively). In addition, we tracked 200 random cells (50 cells per group) manually and calculated the cell cycle duration. Figure 3*a*–*d* shows an example graph of the calculation of the cell cycle duration between cell divisions in the cell trajectories. The average cell cycle durations of the NG, HG, NC-mimics, and miRNA-23a-3p-mimics groups were 14.67 ± 1.87, 16.22 ± 2.0, 15.69 ± 1.68, and 14.38 ± 2.14 h, respectively. The cell cycle duration of the HG group was significantly longer than that of the NG group (*** *p* < 0.001), and the cell cycle duration of the miRNA-23a-3p-mimics group was significantly shorter than that of the NC-mimics group (**p < 0.01; Fig. 3*e*).

### Upregulation of miRNA-23a-3p rescued the HG-induced promotion of apoptosis

**Figure 4.**
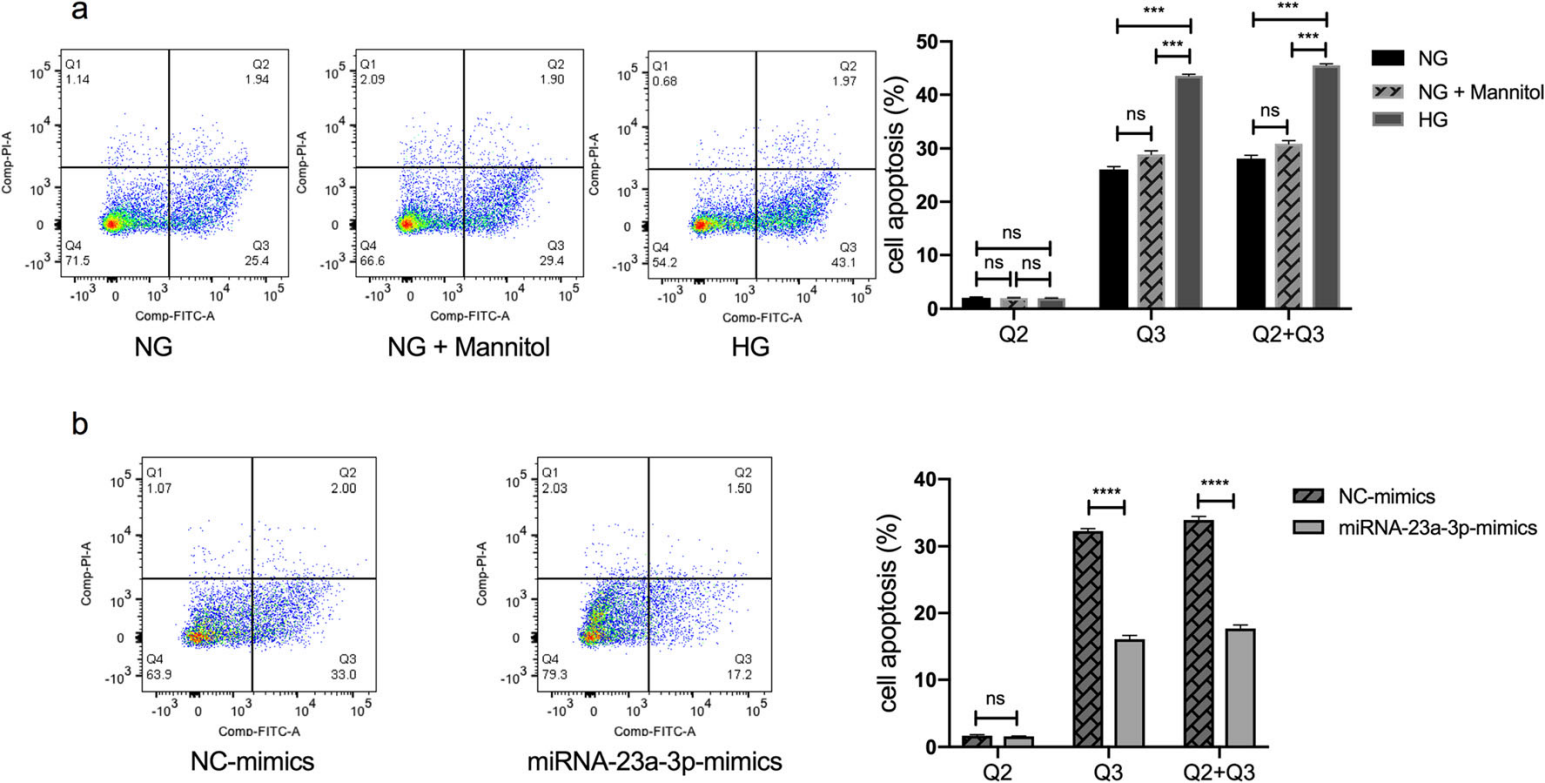
Effects of miRNA-23a-3p overexpression on cell apoptosis in HG-treated H9c2 cells apoptosis analysis by flow cytometry. (*a*) H9c2 cells treated with NG, NG + mannitol (30 mM) and HG. The rates of early cardiomyocyte apoptosis were significantly increased in the HG group (***p < 0.001 vs. NG group). (*b*) H9c2 cells exposed to HG and transfected with NC or miRNA-23a-3p mimics. The rates of early cardiomyocyte apoptosis were significantly decreased in the miRNA-23a-3p-mimics group (****p < 0.0001 vs. NC mimics). FITC fluorescein isothiocyanate, PI propidium iodide, Q2 late apoptotic rates, Q3 early apoptotic rates.

After the cells were treated for 24 h, Annexin V/PI staining and flow cytometry were applied to study the effect of miRNA-23a-3p on H9c2 cell apoptosis induced by HG. The results suggested that HG promoted cardiomyocyte apoptosis (*p* < 0.001; Fig. 4*a*), which is consistent with other studies (Su *et al.* 2019; Yang *et al.* 2019). In addition, as expected, the percentage of apoptotic cells was significantly decreased in the HG-treated H9c2 cells transfected with the miRNA-23a-3p mimics compared with the NC-mimics group (*p* < 0.0001; Fig. 4*b*).

### Upregulation of miRNA-23a-3p affected the expression of cell proliferation- and apoptosis-associated factors in H9c2 cells treated with HG

**Figure 5.**
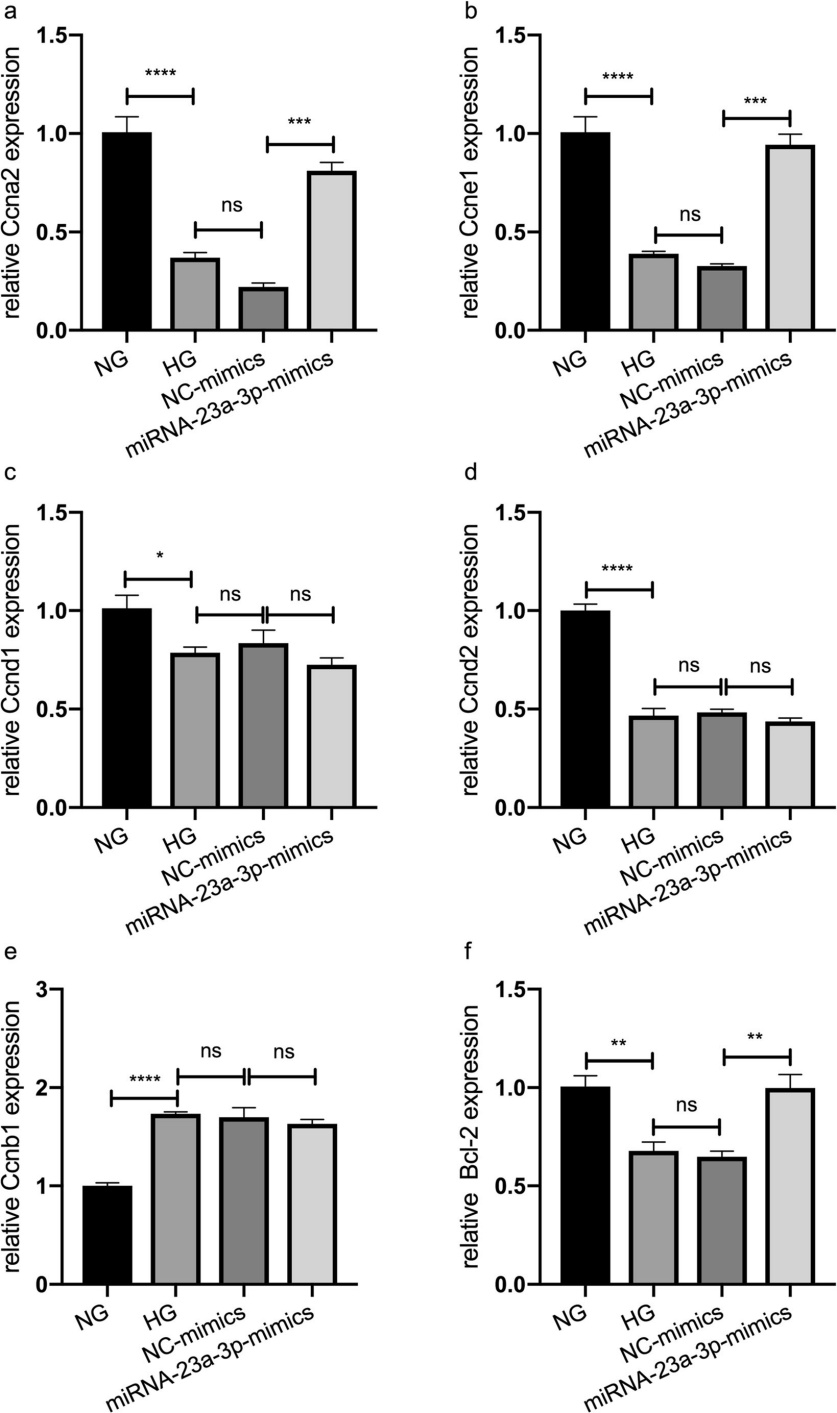
Expression of cell proliferation- and apoptosis-related factors in H9c2 cells under HG conditions and in HG-treated H9c2 cells transfected with miRNA-23a-3p mimics. The mRNA expression levels of *Ccna2*, *Ccne1*, *Ccnd1*, *Ccnd2*, *Ccnb1*, and *Bcl-2* were determined by qRT-PCR. All data are expressed as the mean ± standard error of mean. ns not significant, *p < 0.05, **p < 0.01, ***p < 0.001, ****p < 0.0001 (HG vs. NG, NC mimics vs. HG or miRNA-23a-3p vs. NC mimics).

RT-qPCR analysis was performed to investigate the mechanism of miRNA-23a-3p overexpression in protecting H9c2 cells from HG-induced cell proliferation inhibition and apoptosis, and the mRNA levels of cell proliferation- and apoptosis-associated factors, including *Ccna2*, *Ccne1*, *Ccnd1*, *Ccnd2*, *Ccnb1*, and *Bcl-2*, were detected in each group. The levels of the *Ccna2*, *Ccne1*, *Ccnd1*, *Ccnd2*, and *Bcl-2* mRNAs were significantly decreased in the HG group compared with the control group (*p* < 0.0001, *p* < 0.0001, *p* < 0.05, *p* < 0.0001, and *p* < 0.01, respectively; Fig. 5*a*, *b*, *c*, *d*, and *f*), but the level of *Ccnb1* mRNA was significantly increased (*p* < 0.0001; Fig. 5*e*). However, 48 h after transfection of miRNA-23a-3p mimics in the HG-treated H9c2 cells, the levels of *Ccna2*, *Ccne1*, and *Bcl-2* mRNAs in the miRNA-23a-3p-mimics group were significantly increased compared with those in the NC-mimics group (*p* < 0.001, *p* < 0.001, and *p* < 0.01, respectively; Fig. 5*a*, *b*, and *f*). The expression levels of *Ccna2*, *Ccne1*, and *Bcl-2* were reversed after transfection; however, the expression trends of *Ccnd1*, *Ccnd2*, and *Ccnb1* were consistent before and after miRNA-23a-3p mimics transfection (Fig. 5*c*, *d*, and *e*).

### Upregulation of miRNA-23a-3p restored the proliferation of H9c2 cells treated in HG medium in a dose-dependent manner

**Figure 6.**
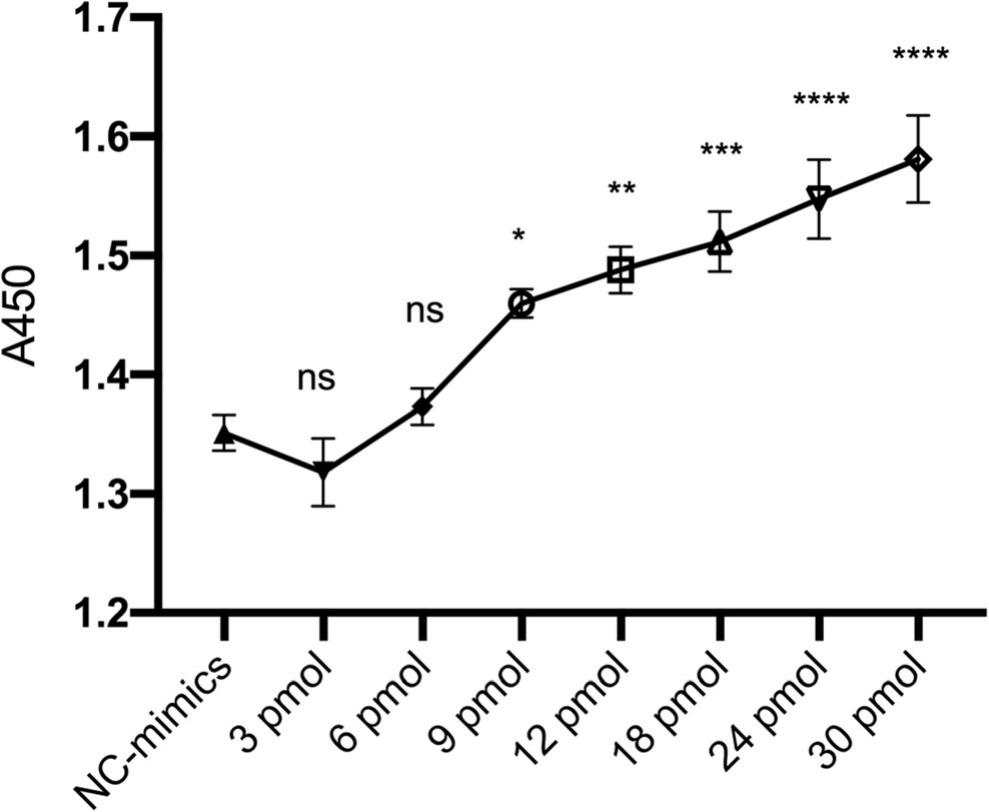
The rescue effects of different doses of miRNA-23a-3p on HG-induced cell proliferation HG-treated H9c2 cells transfected with NC mimics and different doses of miRNA-23a-3p mimics (3, 6, 9, 12, 18, 24, and 30 pmol/well). Cell viability was determined by a CCK-8 assay. ns not significant, *p < 0.05, **p < 0.01, ***p < 0.001, ****p < 0.0001 vs. NC mimics.

miRNA-23a-3p had a protective role in the HG-treated H9c2 cells according to the above findings, but we questioned whether the degree of protection differs at different doses. Thus, HG-treated H9c2 cells were transfected with different doses of miRNA-23a-3p mimics (3, 6, 9, 12, 18, 24, and 30 pmol/well) to investigate the dose-dependent effect of miRNA-23a-3p overexpression on HG-treated H9c2 cells (Fig. 6). The viability of H9c2 cells was tested after transfection with different doses of miRNA-23a-3p mimics, and the results showed that cell viability increased at high doses, but the changes were non-linearly related to the miRNA-23a-3p mimics dose. The 3-pmol/well and 6-pmol/well concentrations had no rescue effect, and the 9-pmol/well concentration started to show a rescue effect.

### The repair ability of miRNA-23a-3p in HG-treated H9c2 cells depended on the manipulation time

**Figure 7.**
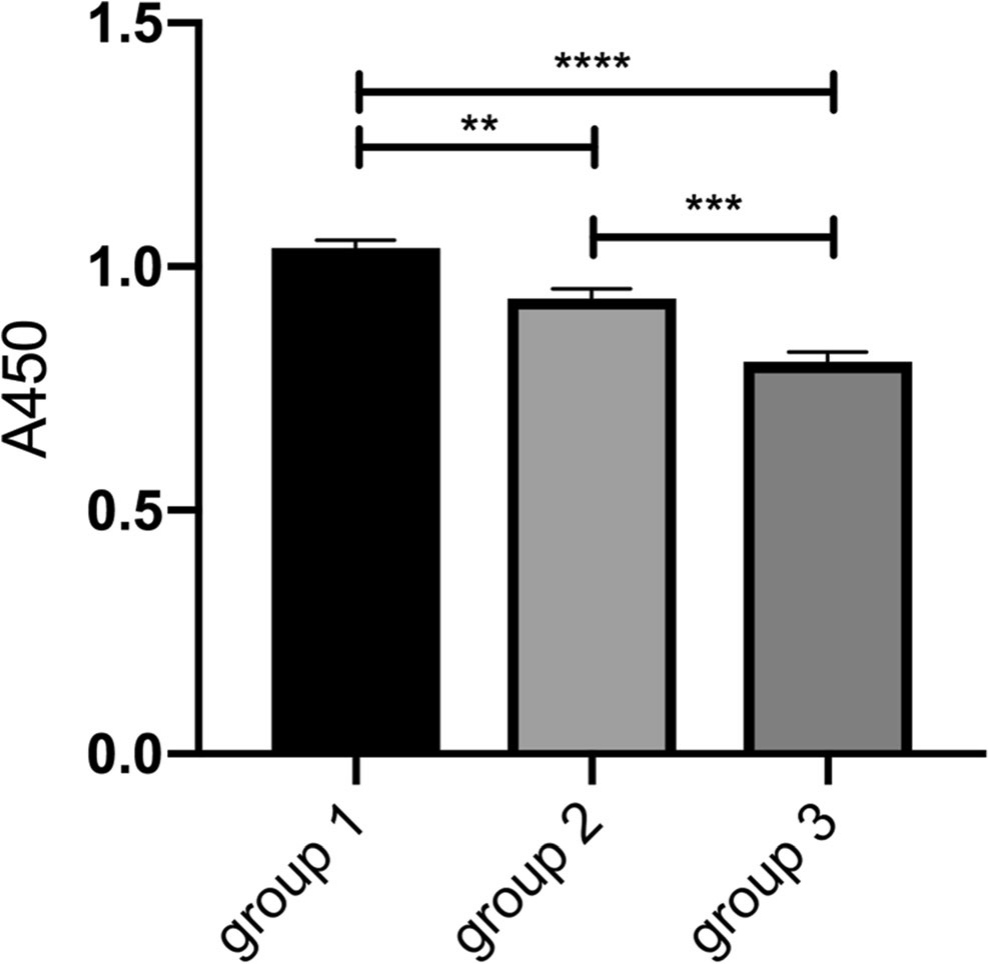
The repair ability of miRNA-23a-3p in HG-treated H9c2 cells depends on the manipulation time H9c2 cells were transfected with miRNA-23a-3p 6 h before HG treatment (group 1), at the same time (group 2), or after 6 h of HG treatment (group 3). Cell viability was determined by a CCK-8 assay. **p < 0.01, group 1 vs. group 2; ***p < 0.001, group 2 vs. group 3; ****p < 0.0001, group 1 vs. group 3.

To explore the effect of miRNA-23a-3p mimics transfection time on the proliferation of HG-treated H9c2 cells, cells were divided into three groups. The first group was transfected with miRNA-23a-3p mimics 6 h before exposure to HG, the second group was transfected at the same time (group 2), and the third group was transfected 6 h after exposure to HG (group 3). Notably, based on the results of the CCK-8 assay, the transfection of miRNA-23a-3p mimics prior to HG exposure increased the proliferation of H9c2 cells compared with the other groups (Fig. 7).

## Discussion

In the present study, we investigated the functional role of miRNA-23a-3p in protecting cardiomyocytes from hyperglycemic injury with a focus on cell proliferation and apoptosis. Our results consistently indicate that upregulation of miRNA-23a-3p may be a method for preventing and rescuing HG-induced cardiomyocyte injury.

miRNA-23 is abundantly expressed in cardiomyocytes, and studies have confirmed that it plays a role in various heart cell types such as cardiomyocytes, fibroblasts, and VSMCs. Both overexpression and inhibition of miRNA-23 have been reported to have a protective effect on heart injury. Overexpression of miRNA-23 could promote the proliferation and inhibit apoptosis of cardiac fibroblasts and VSMCs (Liu *et al.* 2018; Yu *et al.* 2019), while in other studies, the opposite results were observed, where inhibition of miRNA-23 contributed to isoflurane-mediated cardioprotection against oxidative stress (Liu and Liu 2018) and had a protective effect on myocardial ischemia/reperfusion injury in rats (Kou *et al.* 2016). As shown in the present study, upregulation of miRNA-23a-3p rescued cardiomyocyte proliferation inhibition and apoptosis in HG-induced cell injury.

The reasons for the inconsistent results may be due to different cell types, cell environments, and target mRNA expression levels (Hossain *et al.* 2006); since one miRNA may have multiple targets, it may affect different organs or cell types simultaneously. Similar to most miRNAs, miRNA-23 also plays a role in many biological processes and pathways. Although our study demonstrated that miRNA-23a-3p can promote cell proliferation and suppress cell apoptosis in HG-treated H9c2 cells, another study showed that inhibition of miRNA-23 in human mesothelial peritoneal cells could reverse the epithelial to mesenchymal transition induced by HG (Yang *et al.* 2017). These findings support that a single miRNA (such as miRNA-23) can regulate multiple independent pathways, which may converge and lead to a common biological consequence, such as proliferation and apoptosis regulation. Future research can explore whether miRNA-23 can specifically protect against cardiomyocyte damage induced by hyperglycemia in vivo. This study did not determine whether miRNA-23 has a protective effect on myocardial cell damage caused by hyperglycemia in vivo, and future research may help to resolve this issue.

The results of the present research suggest that miRNA-23a-3p can affect cell proliferation and apoptosis by regulating cell proliferation- and apoptosis-related factors. Overexpression of miRNA-23a-3p could rescue HG-induced H9c2 cardiomyocyte proliferation inhibition by regulating the cell cycle, and lens-free microscopy cell data showed that after 48 to 72 h of transfection with miRNA-23a-3p mimics, the number and confluence of HG-treated cells were significantly higher than those in the NC-mimics group. In addition, the cell cycle duration of the miRNA-23a-3p-mimics group was shorter than that of the NC-mimics group. The expression of the cell proliferation-activating factors Ccna2 and Ccne1 was downregulated by HG, but overexpression of miRNA-23a-3p reversed this effect. Ccna2 and Ccne1 have been reported to play crucial roles in the transition of the cell phase from G_1_/S to G_2_/M, thereby promoting cell mitosis (Deng *et al.* 2014; Wei *et al.* 2019). HG treatment can inhibit the transition of H9c2 cells from the G1 to S phase (Zhao and Shen, 2018), and overexpression of miRNA-23a-3p can promote the cell cycle transition from the G1 to S phase by regulating cell cycle-related factors.

The *Bcl-2* family plays an important role in the pathogenesis of cell apoptosis. Previous studies reported that *Notch1* signaling can inhibit H9c2 cell apoptosis induced by HG by regulating downstream apoptosis-related genes such as *Bcl-2* family members (Zhang *et al.* 2015). *SUMO2* overexpression can inhibit the apoptosis of HG-induced H9c2 cells by activating *Bcl-2* (an apoptosis inhibitor) and inhibiting *Bax* and *Caspase-3* (pro-apoptotic factors). Our results also indicate that HG can downregulate the expression of apoptosis inhibitor *Bcl-2* and induce H9c2 cardiomyocyte apoptosis, while overexpression of miRNA-23a-3p activates *Bcl-2*, thereby inhibiting apoptosis. Previous studies (Liu *et al.* 2013; Davargaon *et al.* 2019) have shown that the ratio of early and late stage of H9c2 cell apoptosis changes over time. With the increase of high glucose exposure time, H9c2 cells gradually changed from the early stage of apoptosis to the late stage of apoptosis. After 24 h of high glucose treatment, cells were mainly in the early stage of apoptosis, and then gradually enter the late stage of apoptosis with the increase of time, and at 72 h, the late stage of apoptosis was dominant. Our results were consistent with these studies and also showed that early apoptosis was the predominant after 24 h of high glucose treatment. In this study, we chose to study the protective effect of miRNA-23a-3p on cells when treated with high glucose for 24 h (this is the early stage of H9c2 cell damage).

The transfection effect of miRNA-23a-3p was dose-dependent; compared with the low-dose transfection group, high-dose transfection of miRNA-23a-3p significantly increased the cell viability of HG-treated H9c2 cells, which is consistent with many other studies. The biological effects of let-7a-7f and miRNA-17-92 cluster overexpression were also dose-dependent (Shu *et al.* 2012). R.L. Montgomery and colleagues also found that as the amount of miRNA-29b mimics increases, the target genes decrease in a dose-dependent manner in vitro. A higher dose corresponds to higher delivery efficiency of miRNA-29b to organelles in vivo (Montgomery *et al.* 2014). Overexpression of miRNA-221-5p also inhibited porcine epidemic diarrhea virus replication in a dose-dependent manner (Zheng *et al.* 2018). Clinically, Harry L.A. Janssen *et al.* reported that miravirsen (an antisense inhibitor of miRNA-122) reduced HCV RNA levels in chronic HCV patients in a dose-dependent manner (Janssen *et al.* 2013). The results of this study also showed that miRNA-23a-3p has a minimum effective concentration requirement for the rescue effect. The concentrations of 3 pmol/well and 6 pmol/well had no rescue effect, while the concentration of 9 pmol/well started to show a rescue effect.

Studies have suggested that threshold regulation occurs between endogenous miRNA and target genes and has an inhibitory effect on target genes within a certain range. If the target gene exceeds the threshold, the miRNA inhibitory effect is reduced, and increasing miRNA levels can increase the inhibitory effect to several times within the threshold range (Mukherji *et al.* 2011). The regulation of miRNA is related to the relative abundance of miRNA and target gene. An excessively low abundance of miRNA or an excessively high abundance of target mRNA will cause miRNA off-target effects (Arvey *et al.*, 2010). However, the possible side effects of large doses must be carefully considered, and we need to strike a balance between benefits and side effects.

The transfection time of RNA also affects its biological effect. Our study found that miRNA-23a-3p has a more significant rescue effect in the early stage of HG-induced myocardial injury, and that miRNA-23a-3p pre-treatment can enhance the rescue ability of HG-induced H9c2 cells, which is consistent with some studies. A study suggested that transfection with miRNA-186-5p mimics prior to HG treatment ameliorated HG-induced apoptosis in AC16 cardiomyocytes (Jiang *et al.* 2018). Another study reported that pre-treatment with rAAV-miRNA-22 2 wk before treatment with streptozotocin (diabetic model) can reverse cardiac dysfunction in mice. Additionally, transfection with miRNA-22 mimics 24 h before HG treatment can improve the cell viability of H9c2 cells (Tang *et al.* 2018).

## Conclusion

In summary, our results indicate that miRNA-23a-3p plays an important role in protecting cardiomyocytes from HG-induced injury in a dose- and time-dependent manner. This study may provide new ideas and methods for the prevention and treatment of diabetic cardiomyopathy. However, our study is limited to in vitro results and may not reflect in vivo conditions. In addition, side effects must be considered.
